# Menarche and reproductive health in Spanish Roma women from a reproductive justice perspective: a qualitative study

**DOI:** 10.1186/s12978-023-01726-5

**Published:** 2024-02-02

**Authors:** María Félix Rodríguez-Camacho, María José Sanchís-Ramón, Gaby Ortiz-Barreda, Daniel La Parra-Casado, Diana Gil-González

**Affiliations:** 1 Autonomous Federation of Roma Associations of Alicante, FAGA, Alicante, Spain; 2 Department of Community Nursing, Preventive Medicine and Public Health and History of Science, University of Alicante, Alicante, Spain; 3 Department of Sociology II, University of Alicante, Alicante, Spain; 4 Department of Health Promotion and Development, University of Bergen, Bergen, Norway; 5 CIBER of Epidemiology and Public Health (CIBERESP), Barcelona, Spain

**Keywords:** Roma women, Menarche, Reproductive health, Menstrual health, Reproductive justice

## Abstract

**Objectives:**

This study aimed to explore the perceptions of Roma women about their experience of menarche and reproductive health considering the principles of reproductive justice.

**Design:**

Qualitative study based on semi-structured interviews with Roma women ages 18 through 67 in different neighborhoods in the southeast of Spain. Using a thematic analysis, we analyzed experiences related to menarche and menstruation and their significance for reproductive health, the preparation for the phase of menarche and intergenerational support.

**Results:**

The Roma women interviewed shared their approach to the experiences of menarche and menstruation as children in their family environments with a focus on access to information provided by other women in the family and community for reproductive health management. In their discourses we observed that the onset of menstruation supposes a rupture in the public and private spaces of girls and women.

**Conclusions:**

The results of this study suggest that women and girls do not gain access to information that contributes to their reproductive wellbeing through their experience of menarche. Access to resources and skills to manage biological changes in adolescents could contribute to reducing the impact of cultural myths, false ideas and taboos that prevent advocacy and empowerment on issues of reproductive justice.

## Plain language summary

This study shows that the public spaces surrounding Roma women do not provide the tools and resources to promote and maintain their wellbeing and reproductive health after the onset of this important time. Sexual and reproductive health is not addressed during the time of childhood nor adolescence. Roma women lack a framework with an established discourse on reproductive justice, along with the necessary institutional resources. Preparation for and follow-up of the onset of menstruation among Roma women could be a key for their health. These results coincide with qualitative studies carried out in women from different cultural origins, in which menarche and menstruation are experienced as a transcendental fact. In other contexts, similar to Roma women, this stage in women’s health lacks resources and information and an adequate approach from the perspective of reproductive health, which conditions the menstrual, sexual and reproductive health of women across their lifespan. Roma women experience menarche as a significant change in the life of a woman. They describe having lived the process as an abandonment of childhood and the beginning of an adult life, one that is implicitly linked to maternity and couple relationships. The results show that they defend the continuity of childhood among the new generation. Roma women express that they experienced menarche as an event that was traumatic, embarrassing and confusing. Menarche and menstruation are attended to in private and among the family, and masculine figures do not participate. The mother is the only transmitter of information and support during the process.

## Background

Throughout their life cycle, women present specific health needs, all of which suppose exposure to gender inequalities in health and human rights [1]. Despite the advances and international commitments towards achieving equity and equality in health, which would guarantee women bio-psychosocial health, overcoming inequalities in sexual and reproductive health (SRH) continues to be a challenge for the international public health community [2]. Reproductive health is understood as the interaction of social determinants and individual factors, such as gender roles, limited access to resources and conditions of poverty and segregation. In vulnerable populations inequities in sexual and reproductive health are regulated by sexual and biological relationships and by cultural beliefs that interact to product complex realities [3, 4].

In recent years different studies have shown that experiencing early onset of menstruation results in a greater risk of chronic disease in adulthood [5, 6]. Menarche, or the first menstruation, symbolizes an inflection point in which girls and adolescents define new personality traits and a gender identity [7, 8]. Despite the fact that menarche is an indicator of health in women [9], approaching this phenomenon with an ethnic and gender perspective is uncommon[1, 10]. However, knowledge of and information about menstrual health and hygiene could be a key to living fully in each phase of life as a woman [7, 11]. Scientific evidence shows that women begin this cycle in their lives uninformed, with fear, with shame and without prior preparation to manage future biological, physical, psychological and social changes[12]. Menarche supposes for girls a reconfiguration or rupture of the spaces for equal development, including in education, in the family, among peers and above all in terms of girls’ own self-images [13, 14].

In the healthcare environment, menarche has traditionally been approached as a biological expression of the body, with associated physiological aspects such as discomfort, endocrine disruption, anthropometric indicators and other possible problems [15, 16]. However, menarche is typically interpreted as a new phase in women’s lives in which femininity becomes visible, linked to maternity and marriage [11, 13]. Menstrual bleeding is not only a biological fact, it is also a process that is socially constructed based on cultural interpretations and the configuration of social relationships. This social construction conditions the meaning of menarche and the sexual and reproductive rights of women [2, 9] as well as access to the enjoyment of reproductive justice spaces [17].

Therefore, menarche is linked to multiple aspects of women’s lives. These life processes of women represent their abilities and capacity for empowerment in terms of reproductive rights. Ruptures in these processes affect not only the time of menarche but also future relationships and the configuration of their sexuality [18, 19].

Different authors affirm that experiences of menarche and knowledge about menstruation can condition the end of the phase of childhood [20, 21]. In the educational context Roma girls are more at risk of school abandonment, school failure and adolescent pregnancy [22]. There is no available literature that links menarche to spaces where Roma girls interact, however, a study carried out in Malawi showed that age at the onset of menarche was linked to early sexual relationships and premature school abandonment [20].

The first authors that approached reproductive justice (RJ) did so from a holistic SRH perspective, defined as the self-determination that women can exercise over aspects of their lives. Reproductive justice includes choices about childbearing and decisions about raising children. It also includes aspects such as choice of spoken language and dedication to raising a family in a woman’s culture of origin [23, 24]. It is a basic right related to making decisions about one’s own body and receiving the necessary and complete information in all areas related to health and wellbeing [25]. For reproductive justice to exist, women must recognize contextual elements and become empowered in areas that allow them to enjoy their reproductive lives [26]. In this sense, interviewing Roma women provides information about the ways in which menarche is a health phenomenon that marks the end of childhood and conditions their reproductive health and lives.

In Spain Roma women are a population that is characterized by a greater risk of poor physical and psychological health, including poor sexual and reproductive health [27]. In Spain, the Roma population is an ethnic minority, they also self-identify as Calé, Kalé, gypsy or Spanish gypsies. They are largest ethnic minority in the country,accounting for around 1.5 to 2.1% of the total population. Roma women experience triple discrimination due to gender, culture and social status [28]. In 2014, the Roma health survey showed that Roma women have less access to essential women’s health services, such as visits and gynecology consults, and prevention of breast cancer and cervical cancer [29, 30]. Studies have shown that sexual and reproductive health is the area in which the most discriminatory practices against Roma women take place [31, 32]. Within their families, these women are affected by a patriarchal structure that is supported by and operates through a social context of discrimination and structural violence [27, 33]. Traditionally defined roles control not only rebellion and transformation, they also generate gender inequalities and a double discrimination expressed not only in the health of Roma women but in all of the dimensions of their lives [27, 33]. Roma women present higher rates of school abandonment, higher unemployment and greater dependence on social services. Control over Roma women’s reproduction represents a lack of reproductive justice. Forced sterilization experiences in Europe and family planning programs are examples of abuse of reproductive rights and the lack of visibility and social representation of Roma women in the public sphere [34, 35].

Given that principles based on reproductive justice are key for decision making about women’s lives and bodies, the topic of menarche, as a key stage in women´s life experience of women, could contribute to the promotion of sexual and reproductive health among Roma women, not only in adolescents but throughout their lives [1, 36]. Currently, there is no known scientific literature with an ethnic and gender focus that concerns reproductive health in Roma women. Thus, this study aims to explore the perceptions of Spanish Roma women about menarche and sexual and reproductive health with a reproductive justice focus.

## Material and methods

### Design and context

We carried out a qualitative study based on semi-structure interviews with Roma women in the province of Alicante (Spain). To collect information, neighborhoods were selected from municipalities with large concentrations of Roma population. This study was carried out under the framework of the European Project Empowering Roma Girls' Mattering through Reproductive Justice [RoMOMatter] between 2019 and 2021. The project involved the participation of Roma communities, local organizations and scientific institutions in Bulgaria, Romania and Spain. The project involved a participatory empowerment intervention among young Roma girls that used a gender perspective to contribute to building tools to support making informed decisions about their own lives [28].

### Study population

Roma women were interviewed who were residents of the selected municipalities in Alicante (10), Elda (4), El Campello (2) and Almoradi (3). It was considered that this provided enough diversity to obtain narratives that would provide different visions and perspectives about sexual and reproductive health. Women were contacted through the collaboration of local Roma organizations. A total of 19 women ranging in age from 18 to 67 were interviewed. Information was collected on primary characteristics such as age, number of children, marital status, employment situation and level of education (Table 1).

**Table 1:** Sociodemographic characteristics of the participants

ID	Age	Children	Civil status	Job	Level of education
01	24	0	Single	Employed and studying	University
02	28	Pregnant	Married	Housewife	Primary school
O3	38	3	Married	Employed	Unfinished primary school
04	33	4	Married	Housewife	Unfinished primary school
05	56	4	Married	Employed	Unfinished primary school
06	28	4	Married	Studying	Unfinished primary school
07	30	3	Married	Housewife	Unfinished primary school
08	67	4	Married	Housewife	Illiterate
09	39	3	Married	Employed and studying	Secondary (high school)
10	32	4	Married	Employed	Secondary (high school)
11	21	1	Married	Housewife	Unfinished primary school
12	43	3	Married	Housewife	Unfinished primary school
13	53	0	Single	Employed	Primary school
14	22	0	Single	Unemployed	Primary school
15	21	0	Single	Unemployed	Secondary (high school)
16	29	3	Married	Housewife (looking for a job)	Primary school
17	18	0	Single	Studying	University
18	55	1	Single	Employed	Unfinished primary school
19	20	0	Single	Studying	University

### Instrument and information collection

An interview guide was designed with 43 topics/questions, organized into eight thematic blocs that included the following sections: open questions, menarche stage, mothering, empowerment, mattering, family planning, reference persons, closing questions. These were areas of interest to the RoMOMatter (www.romomatter.org) project. In this study we focus on the results from block number two, which explored the perceptions, experiences, needs and sociocultural changes associated with menarche and menstruation in adult Roma women.

### Information organization

Nineteen interviews were carried out between April and June of 2019. All of the participants were informed about the study, both orally and in writing, and signed an informed consent document. Two of the authors carried out the interviews: both participated in information collection, transcription and later analysis. The two interviewers were women, one was of the Roma ethnicity and the other had family relationships with the Roma community, which created an environment of empathy and trust as well as knowledge of cultural cues.

For each women interviewed, we required a prior appointment (in-person or via phone). The interviews were carried out in places that guaranteed privacy, without the presence of other people. Participation was voluntary, and the women were not compensated for their participation. The interviews lasted between 30 and 60 min.

### Analysis

This study followed the six steps of thematic analysis described by Braun and Clarke [37]. Interviews were recorded digitally and later literally transcribed. Audio recordings were reviewed and matched with interview codes that were assigned to anonymize them. Field notes and observations were also reviewed for each interview. A list of themes was developed prior to the interviews, and the first coding consisted of classifying the responses into these themes (Table 2). Two authors combined the detailed readings of all of the transcriptions and identified the parts of the interviews related to experiences of menarche and reproductive health among Roma women. In the next inductive step, codes were generated for fragments of text that emerged based on the treatment of the information. A random sample of interviews was coded by another member of the project research team, and the findings were compared as a group. Categories were derived from the grouped codes, and an analysis strategy was proposed to produce the preliminary themes. The members of the research team double-checked all of the codes and contributed to the code categorization and grouping. The analysis software ATLAS Ti 8 was used to facilitate the process of organizing the information and coding.

**Table 2:** Topics considered in block 2: menarche

1. Meaning of menstruation: How the interviewee felt when she had her first period and what it meant to her
2. Meaning of the relevance of menstruation: General meaning and social relevance of the first period, the change from girl to woman (“becoming a woman”), etc
3. Meaning of girls’ preparation for the first menstruation: preparation for menarche: Who prepares girls for this moment? How should girls prepare themselves? When should they prepare?

### Validity and reliability

In terms of validity and reliability, the process was guided by 1) transparency (different researchers from among the project team members provided verification, and furthermore, the data were a part of a European study and could be validated; 2) communication (the categories had to make sense for the researchers, and researchers were consulted every step of the way), and 3) coherence (the categories had to be internally consistent and at the same time reflect both individual differences as well as genuine inconsistencies in the discourses).

## Results

**Fig. 1:**
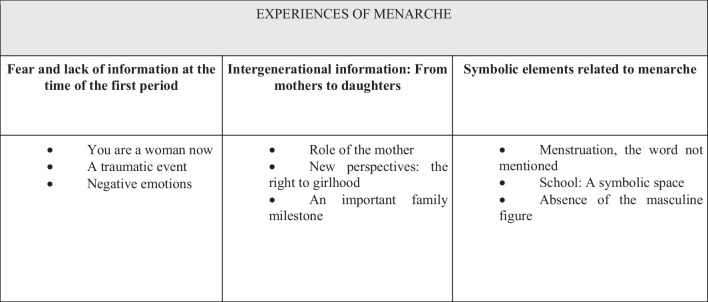
Themes and sub-themes extracted from the analysis

Three themes were identified related to menarche and the experiences of Roma women, including: (1) fear and lack of information at the time of onset of menstruation, (2) intergenerational information on menarche: from mothers to daughters, and (3) symbolic elements related to menarche. Figure 1 shows the sub-themes for each theme.

### Fear and lack of information at the time of onset of menstruation

#### Now you’re a woman

The findings suggest that menarche is an experience that is accompanied by fundamental and transcendental changes in the lives of the interviewed women. They described surprise, lack of prior knowledge and the mother figure as a reference for such knowledge. Menarche was experienced repeatedly as the conversion and transition into "being a woman,” in which the lack of information resulted in descriptions of “crying," “trauma," “giving in” and "blocked". Menarche was translated into the transmission of different gender ideals, responsible attitudes and formal changes in behavior and health, associated with the confusion of what is feminine and permitted or prohibited.

The first time? I cried and everything. I was just a girl, and I cried “mama, what is this? I’m bleeding!” My mother told me I was a woman, and that was it. In the beginning it was really hard for me, because I was a girl and couldn’t go to the swimming pool or anything, and I cried a lot. But in the end, you get used to it. (E14, 22 years)

I was completely embarrassed, and I suffered from trauma, I really did, about it [menstruation], because I was only 9 years old (…) my mother said, “Ay, now I’ve got a woman”. I suffered trauma, yes. Later, of course, I adapted, time passed. (E03, age 38)

You have to be a woman and have more responsibility, and of course, the first time you have it [menstruation], you don’t know what this responsibility is. (E04 age 33)

Yes, (…) you proceed with this (…) caution, like they told you: “Well, wow, you now you need to be careful," like my mother used to say, “You can’t play with boys anymore…” I didn’t understand it. It’s like something lights up in you, “Hey, careful,” they say, or you say, “This, I can’t do it,” for example riding a bike, I used to love riding a bike (…), “Girl, get down off that bike (…) that’s for men, you can’t ride a bike!” I used to like to ride bikes so much that I would ride it in secret (…). The truth is that you think, “I can’t ride a bike anymore, I have to be more careful (…). (E-18, age 55)

#### A traumatic event

The women describe menarche as a traumatic experience in which they begin to suffer and they stop recognizing themselves. These moments are painful, both at the physical and the mental level. They remember the time period of menarche in terms of the sensation of being vulnerable due to lack of knowledge**,** solitude and fear.

I said, my goodness for me it was a trauma, a trauma you know? My mother felt really bad too, I was very young [10 years old]. (E 06, age 28)

You suffer physical pain, but you also suffer at the emotional level, I think. You’re more sensitive, the way you relate to people. You change, when your menstruation begins, you change. One might suffer physical pain but she also might not recognize herself. (E01, age 24)

My mother was always out working, so I didn’t have anyone to support me. For me it was a little traumatic, in the sense that I hid it from everyone, no one knew about it. (E05, age 56)

#### Negative emotions

The participants express negative emotions that brought up feelings of fear, suffering and resignation. Developing negative traits and emotional qualities such as shame and discomfort is at the root of the silence and disorientation expressed in the interviews. Menarche and menstruation are experienced as an intimate and private secret, accompanied by feelings of terror that can paralyze and confuse girls.

I went to the bathroom and when I saw that everything was stained red I got scared, really scared, and I said, “Wow, what is this? Did I cut myself? I looked at myself everywhere to see where I might have cut myself, and then I cleaned myself up and I went, scared, to my seat at school. I didn’t say anything to the teacher, and I was scared. Nor did I say anything to my mother. I kept it inside. (E15, age 21)

No one ever said anything about it. I was 11 years old at the time of my first period (menstruation), and I realized when I went to the bathroom and started bleeding. I got scared, and at the same time I stayed silent. I didn’t tell anyone because I didn’t know what it was, and I didn’t want anyone to know about it. I didn’t trust my mother, she had never mentioned menstruation, so how was I going to tell her? I was bleeding a little bit scared. (E18, age 55)

### Intergenerational information on menarche: from mothers to daughters

#### The role of the mother

Menarche and menstruation are family health issues, the responsibility for which fall to the mother figure. In their role as transmitters and references, they share different opinions. The mother is the primary source of information for girls, with whom girls most closely identify. Emotions related to menarche are re-experienced with daughters, replicating emotions of fear, confusion and what is permitted and prohibited. The participants expressed that the information they received about menarche as children was scarce and inadequate. Some participants consider menarche to be an understanding of the body linked primarily with sexuality and being a woman. Having conversations with their daughters about these health topics makes them repeat patterns of disinformation or of flight and evasion. The sense of responsibility of the women for their daughters, involving initiation of intimate relationships (in the interviews the term “marriage” was avoided), means that the participants reject any additional information about menstrual health and thus prolong their daughters’ childhoods. They coincide in the fact that there is currently insufficient information on reproduction and menstrual health, although it is more than the received themselves.

It has to be the mother [referring to who is responsible for preparing girls in terms of sexuality and menstruation issues]. Right? That’s what I say. It’s not the same if you tell your father, or someone at school. This is the responsibility of the mother; your family needs to explain it to you. (E10, age 32)

No, she didn’t tell me beforehand. And I said, “Mom, what is this?”, and she said “When we become women, this is what happens to us [referring to menstruation], we bleed and get stained…” I said, “Wow Mom, blood?” And she said, “Yes, when this happens to you, you have to come and tell me.” (E14, age 22)

I have a 10-year-old daughter, and I don’t know…I don’t know what to say to her, because for me my daughter is still just a girl. The day she becomes a woman, for me she’s still going to be my daughter. It’s what my mother told me, that I was still her little girl…of course. (E12, age 43)

I feel responsible. It’s very difficult, very difficult. You [referring to herself] are young and have had a daughter very young. I have a lot to learn, and now I need to teach her. It’s very complicated. I make that might be bad, because I don’t know how to manage her [referring to her teenage daughter], and this is scary. (E06, age 28)

I don’t dare to tell her anything. I’ve never sat down to tell her. I think they already know more [about sexuality], much more than we think they do. Because of the television, how things are, the mobile phones. (E03, age 38)

I think it’s not necessary for a girl of 8 or 10 years to be talking about these things [sexuality], what it means to be a woman in these times. Of course, we Roma people, we have this respect, this idea that until you’re married you won’t do anything [have sexual relationships]. Because if you start at age 11 or 12 (…). There are girls who, after two or three days, want to get married [initiate sexual relationships]. They want to go and destroy their lives. Since I don’t want this, well, I try not to talk about it. (E04, age 33)

#### New visions: The right to girlhood

On the other hand, for the women menarche was related to the beginning of a phase of learning, responsibility, caregiving and a premature adult life, which they didn’t want for their daughters. The participants mention that to be a woman is to represent the worst part of society, in which “only women have to suffer”. Therefore, new visions emerge as well as advocacy for their daughters and their rights to girlhood, or to “be a girl”. The women coincide in that Roma culture is not external to the cultural and generational changes of the environment. The narratives describe prolonging the life phase which in prior generations was ended by menarche. This prolonging of childhood implies the possibility of another life phase (youth) which did not appear in the prior generation.

I don’t like to talk about it with her. The date she got it [her period] I told her that she was getting older, that it wouldn’t be the same as before, but I scolded her not to become a woman. She’s a girl and I didn’t want to deprive her of girl things, and around 13 it would be time to become a woman, to grow up, have her own worries and bear the things in life that we woman always have to bear (E04, age 33)

To me she seemed so young, such a girl, because she had just turned 11 and was still going to elementary school. She was so young, for example, being 11 for her was not the same as when I was 11. I was more mature, I could stay alone with my nephew to take care of him, change his diaper, bathe him, feed him, in other words, care for a baby. But I didn’t see my 11-year-old daughter as capable of this (E09, age 39)

No, she doesn’t think about lipstick or mascara, I like the way my daughter things. You know what she thinks? She wants to study a university degree. She thinks about studying, and I support her in this (…), she says herself that she’s only going to have a childhood once, so she has to value it. She has to be a girl (E12, age 43)

That’s why I’ve always done it, in other words, they didn’t do it with me. With my daughter, I’ve made this process be more natural. Just because you get your period doesn’t mean you’re more mature, that you’re a woman and you stop being a girl. You keep on with your life and your maturity comes with age (E09, age 39)

I got emotional because I felt that she was growing up [menarche], and it felt sweet. Unlike me, I had already explained it to her. Let’s treat it as normal, since it’s normal (…), I’m teaching her this, to prepare for this life, to study, and that there is time to marry later. They have a lot of time (E10, age 32)

#### An important milestone for the family

For the family and the environment, menarche and menstruation are relevant and lined to marriage and motherhood. Menarche is considered a risk factor for embarking prematurely on adult life, which supposes an end to the stage of childhood. In terms of information shared in families, conversations are incomplete, which ends up perpetuating fears and prejudices around gender and ethnic roles.

I think this is like menstruation. Menstruation is not taught much among Roma people. They don’t teach you; they tell you more about marriage [sexual relationships] than about menstruation (E19, age 20)

What does a woman’s first period mean? Maybe parents get scared because girls can get pregnant and are growing up, and unfortunately there are still many people, many girls who get married early. Maybe they say, “oh, already?”, like they protect them more, they don’t let them go out, or just to buy bread and that’s it (E10, age 32)

The life of Roma people who have a daughter between 14 and 15 years old, you always go around with that worry that she will find someone to be her boyfriend and leave you early. So well, you’re always keep an eye out, saying, “Don’t go with them, nor with the others.” (E05, age 56)

Because Roma women marry so young, that is to say, now [refers later to menarche], if you’re with a man well you’re going to get pregnant (…). There are people that say, “you got your period”, it’s for the family, so it’s a rough change from girl to woman, but no, she’s still just a girl (E09, age 39)

### Symbolic elements of menarche

#### Menstruation: the word is not used

For the participants menarche signifies a biological change surrounded by cultural and social beliefs that condition the health and life of Roma girls and women. The information on menarche is transmitted in a way that is scarce and biased, and in the majority of case, the term is linguistically modified and referred to as “this” or “that”. Women pass these messages about femininity to other generations that also perpetuate them. Menstruation is an inheritance from woman to woman transmitted in an incomplete way. As a sign of respect, the participants do not use the term menstruation, and they prefer to use symbolic and metaphoric examples to refer to the menstrual cycle.

I so talk to my grand-daughter, to all of the girls and my young girl. I have told her that when you get the sartenero [menstruation] you’ll be dirty all over. (E08, age 67)

I don’t know, I think it was the moment when (laughs embarrassed) you have, you have, should I say it? [Interviewer: “Of course.”] When you get your first menstruation, I think that, in that moment, although I got it when I was 11, at that moment it is your mother who prepares you. E14, age 22)

I remember that my father said, “Congratulations”. He gave me a kiss and so on, but really you don’t know what is going on (….). My grandmother is someone who can’t talk about it in front of men (…). You can’t talk about that topic (…), it’s unattractive (…). She doesn’t talk about that subject. (E01, age 24)

#### School: a symbolic space

The participants describe school as a space that is not susceptible to culture and lacks cultural competency to manage sexual or relationship education without judging adolescents for being Roma. School is associated with girlhood, and menstruation is understood as an abandonment of girlhood. In this sense, school becomes a hostile environment for Roma girls, given that it creates and reaffirms stereotypes regarding the attitudes and behavior of girls. In the minds of the participants, the time that passes from the onset of menarche to the early adult life is short. According to the women, they married very young and become mothers between ages 14 and 20. One of the participants relates her experience being an adolescent mother at school. After becoming pregnant she had to permanently abandon her studies.

Girls feel embarrassed and uncomfortable when they talk about these things. They talked about it at my school, and I felt ashamed. I don’t see how they talk about it, with boys there, as the schools do. I know it is natural, but I don’t understand it, for Roma girls. Because when I went to high school, they gave sexual education and told us all about women and all about men [refers to reproductive organs] and we died of embarrassment, and me even more. I don't know why they talk about these things (E07, age 30)

It’s all the same to me, whether they do it at school of not. But I think they don’t give them these classes and they don’t explain. They don’t explain the day to day of what can happen, or the consequences of a relationship that they can have (…). Not at the schools. (E19, age 20)

I was married and I went to high school because I was dedicated [refers to being a good student] (…). Up until the day they found out I was pregnant, and the school didn’t let me go back. (…). Because they didn’t want to be responsible for what could happen to me inside, because a child could push me, they didn’t want to be responsible for me (….). Of course, I wanted to have my home, my family, my children, but I wanted to achieve something more with my life, to be able to study, even if only to graduate, but, well, that’s as far as it went. (E11, age 21)

#### Absence of a masculine figure

The absence of a father figure on questions related to menarche translates into certain ideas about the masculine figure that should be shielded from this part of femininity.

Well, of course we have to change, but I have to hide all the time so people don’t see because it’s not pretty [menstruation]. My father doesn’t like it, and if sometimes we leave something there and it’s a bit visible, he’ll say “What’s this [refers to a menstrual pad]!” (E17, age 18)

I got very sick (…). Then my father asked, with all of the trust of a father, that I tell him everything, but I never did, not to my brother, nor to my father. It was out of respect, and I felt embarrassed. But my father has always been, as well as my mother, a man and a woman that you can trust with everything. My brother and the little ones trust them. In my case I felt embarrassed, because those were other times. (E02, age 28)

## Discussion

Roma women experience menarche as a significant change in the life of a woman. They describe having lived the process as an abandonment of childhood and the beginning of an adult life, one that is implicitly linked to maternity and couple relationships. The results show that they defend the continuity of childhood among the new generation. Roma women express that they experienced menarche as an event that was traumatic, embarrassing and confusing. Menarche and menstruation are attended to in private and among the family, and masculine figures do not participate. The mother is the only transmitter of information and support during the process.

This study shows that the public spaces surrounding Roma women do not provide the tools and resources to promote and maintain their wellbeing and reproductive health after the onset of this important time [38]. Sexual and reproductive health is not addressed during the time of childhood nor adolescence. During menarche Roma women experience a reconfiguration of the role of women and feel limited and inhibited our to the fear of feeling exposed and judged in a social context without representation of women and Roma. They have, for example, attitudes of fear of being discovered during menstruation or fear of staining their clothing. Silence is a common response to the situation, which leads to a lost opportunity during the period of monarch: missing out on information, the capacity to choose and the right to exploring their sexuality and reproductive health without discrimination or violence [39]. These results coincide with qualitative studies carried out in women from different cultural origins, in which menarche and menstruation are experienced as a transcendental fact. In other contexts, similar to Roma women, this stage in women’s health lacks resources and information and an adequate approach from the perspective of reproductive health, which conditions the menstrual, sexual and reproductive health of women across their lifespan [26, 41, 45, 46].

Menarche represents the first point of contact with reproductive possibility [10]; however, disinformation, silence and feelings of vulnerability predispose Roma women not to become empowered to defend their reproductive rights nor those of their daughters [1, 11, 22]. In this study, Roma women describe the onset of menstruation as occurring at early ages of between 9 and 12. This fact often prevents access to adequate information, given that the women do not consider their daughters to have sufficient maturity to digest information on sexual and reproductive health [40]. However, our study shows the existence of two forms of generational transmission -from mothers to daughters. On one hand, some mothers repeat patterns of disinformation and fear related to beliefs about their bodies and menstrual health. For these women, menarche is broadly related to maternity and the adult responsibilities of a woman [41]. In contrast, new visions emerge among other women, in which menarche is not interpreted as an abandonment of childhood and the beginning of adulthood, rather they advocated for the right of their girls to experience girlhood. From this perspective, women defend the experience of natural processes and protection of youth as a life phase among new generations. This position coincides with a study by Sommer (2013), which shows the importance of preparing girls properly for the onset of menstruation, given that it is a key moment in education and empowerment in the area of sexual and reproductive health [42, 43].

In this study, Roma women hide and remain silent, and live this part of their lives in the most private possible way. They have not internalized reproductive rights or information that protects them as a social group [41, 44]. In other studies, reproductive justice is a key for women in recognizing their reproductive rights, which reduces discrimination based on ethnicity or culture [25, 45]. For a movement such as reproductive justice, it is necessary that women have access to information to advocate for their reproductive rights [23]. Interpreting the results of this study under the principles of reproductive justice widens the vision of SRH among Roma girls and women towards a more holistic approach of health and wellbeing, including dimensions of life such as culture, society, religion and gender roles [17, 46].

Gender is a principal health determinant that generates biases and inequality [47, 48]. Menarche and menstruation are life processes of interest for public health. Women’s health requires analysis from the perspective of gender and attention that is focused on women. The work must be carried out with a focus on the realities of women’s daily life and experiences [36, 49]. Including men in education on menarche and menstruation is fundamental for promoting a focus on equality. The scarcity of participatory actions (from research to intervention) related to knowledge of resources and tools related to SRH among Roma women and girls are contrary to reproductive justice principles [22]. Approaching SSRH among Roma women must be based not only on the reduction of early pregnancy and family planning, it also must increase awareness in Roma women’s environments in order to empower them to make decisions about their bodies [50, 51].

Reproductive justice is also related to intercultural abilities as a strategy to orient public policy interventions to reduce chronic diseases in addition to early pregnancy. Intercultural abilities are essential tools for health and social education to improve sexual and reproductive health among all women [22, 33]. Incorporating these abilities and competences in the development of policies is a step towards an approach based on reproductive justice.

Evidence suggests that this process in women’s lives is not exclusive to Roma women [1, 42], however, we did not find literature on this topic related to advocacy on menarche and reproductive justice among women. Given the link between the age of onset of menstruation [20] premature school abandonment and early pregnancy, it is necessary to continue this area of investigation. The qualitative approach of this study provides unique knowledge on the reality of women and their experiences with monarch. Their expectations and experiences generate an understanding of the phenomenon studied in all of its personal and community dimensions. However, this study has limitations. The participants and interviews were located in a single province where the European RoMOMatter Project was carried out. This could have limited the access of women with other realities that were relevant for this study. However, the diverse profiles of the interviewed women offers adequate representation of Roma women’s perspectives on menarche. On the other hand, it should be noted that the discourses in this study were collected in an interview that also addressed other topics. There was a section that addressed menarche. Although the information collection instrument was not exclusive to menarche, the questions contained were strictly reviewed by the research group. The fact that the interviewed women were Roma people (as was some of the research team) also facilitated the access to the interviewees.

## Conclusions

In conclusion, for Roma women menarche is a relevant phenomenon that affects sexual and reproductive health. Menarche is experienced with fear and in solitude, and although mothers are the principal source of information, there is a lack of information and stigmatizing approach to this issue. Roma women lack a framework with an established discourse on reproductive justice, along with the necessary institutional resources. Preparation for and follow-up of the onset of menstruation among Roma women could be a key for their health. There are currently no tools related to the promotion of (or actions related to) reproductive health to improve accessibility, quality, and use of services with a reproductive justice focus. Public policy also lacks such a focus. Public health must take into account the future monitoring of menarche as an important milestone in the lives of women and as a facilitator for improving their menstrual, sexual and reproductive health. It is important to include an intersectional perspective in reproductive healthcare for Roma women. These women lack environments that emphasize reproductive health from childhood throughout adolescence. Therefore, it is essential that they be able to develop their rights in environments without violence or inequality.

## Data Availability

The availability of data on this project can be found on the website https://romomatter.org/toolbox/**.**
